# Antineutrophil cytoplasmic antibody-positive familial Mediterranean fever and hyperthyroidism: A case report

**DOI:** 10.1097/MD.0000000000013805

**Published:** 2018-12-21

**Authors:** Sorato Segoe, Ken-ei Sada, Keigo Hayashi, Yuriko Yamamura, Michiko Morishita, Haruki Watanabe, Yoshinori Matsumoto, Jun Wada

**Affiliations:** a Okayama University Medical School; b Department of Nephrology, Rheumatology, Endocrinology and Metabolism, Okayama University Graduate School of Medicine, Dentistry and Pharmaceutical Sciences, Okayama, Japan.

**Keywords:** antineutrophil cytoplasmic antibody, familial Mediterranean fever, periodic fever, pleurisy, propylthiouracil

## Abstract

**Rationale:**

Familial Mediterranean fever (FMF) is a genetic autoinflammatory disorder characterized by serositis and recurrent fever. Previous reports identified patients with antineutrophil cytoplasmic antibody (ANCA)-positive FMF, but vasculitis symptoms were not reported.

**Patient concerns:**

We report the case of a 44-year-old man with numbness. He had a history of 3 episodes of pleurisy and was being treated with propylthiouracil for hyperthyroidism. Because he was ANCA-positive, we suspected drug-induced ANCA-associated vasculitis and propylthiouracil was discontinued. However, his numbness was not ameliorated, and he again developed high fever with pleurisy.

**Diagnosis:**

Diagnosis of FMF was finally made, and genetic analysis revealed compound heterozygous mutations in exon 2 of the *familial Mediterranean fever* gene (L110P/E148Q).

**Interventions:**

The patient was treated with 0.5 mg/day of colchicine.

**Outcomes:**

His numbness improved, and fever has not recurred.

**Lessons:**

Appearance of ANCA and development of vasculitis should be considered in a clinical course of FMF with hyperthyroidism.

## 1 Introduction

Familial Mediterranean fever (FMF) is an autoinflammatory disorder characterized by serositis and recurrent high fever. In typical cases, fever lasts for 1 to 3 days, and abdominal and chest pain are caused by serositis. Recurrent inflammation may result in secondary amyloidosis, but administration of colchicine improves the symptoms in over 80% of patients with FMF.^[1]^ FMF is a relatively rare disease with autosomal recessive inheritance and *familial Mediterranean fever* (*MEFV*) gene mutations in exons 10 and 2.^[2]^

Antineutrophil cytoplasmic antibody (ANCA) is an autoantibody against antigens present in the neutrophil cytoplasm and is associated with a form of small vessel vasculitis known as ANCA-associated vasculitis (AAV). It is also well known that propylthiouracil (PTU) induces ANCA production. In fact, it has been indicated that the abnormal conformation and impaired degradation of neutrophil extracellular traps (NETs) induced by PTU leads to ANCA production and AAV.^[3–5]^ Other reports have showed that patients with FMF were ANCA-positive and that NETs were related to the activity of FMF.^[6–8]^ Here, we report a case of ANCA-positive FMF with numbness during PTU treatment.

## 2 Case report

A 41-year-old man presented to a local hospital with upper and lower extremity motor disturbances in 2014. Based on a mild reduction in nerve conduction velocity, he was diagnosed with polyneuropathy. In 2016, he experienced 3 episodes of pleurisy with fever (body temperature >38°C) at 2-month intervals. Because he was both myeloperoxidase (MPO)-ANCA and proteinase-3 (PR3)-ANCA-positive, he was referred to our hospital in June 2017. His family history was unremarkable and he had no history of drinking. However, the patient had smoked 20 cigarettes per day for 25 years. His medication included 50 mg/day losartan and 5 mg/day amlodipine for hypertension. Since 2006, he had also been treated with 100 mg/day PTU for hyperthyroidism. His laboratory test results during the first visit were as follows: white blood cell count, 3980/μL; C-reactive protein (CRP), 1.39 mg/dL; MPO-ANCA, 10.20 EU/L; and PR3-ANCA, 6.76 EU/L. PTU-associated AAV was suspected at that time, and PTU was discontinued. After discontinuation of PTU, CRP levels normalized, but the numbness did not improve.

In November 2017, he was admitted to our hospital with chest pain and high fever. His body temperature was 37.8°C and blood pressure was 158/109 mm Hg. His consciousness was clear, and he had no signs of neurological, respiratory, or abdominal involvement. Pleural friction rubs were not heard. Manual muscle strength testing was normal. Laboratory findings on admission were as follows: white blood cell count, 6760/μL; CRP, 8.65 mg/dL; creatine kinase, 512 U/L; PR3-ANCA, 6.88 IU/mL; and MPO-ANCA, 12.7 IU/mL. Computed tomography showed longitudinal enlargement of the mediastinal lymph nodes and pleural effusion, indicating pleurisy.

After admission, his fever and chest pain improved, and CRP levels decreased spontaneously. As his clinical course was consistent with a major Livneh criterion (typical attack with unilateral pleuritis), he was diagnosed with FMF.^[9]^ Genetic analysis also revealed compound heterozygous mutations in exon 2 of the *MEFV* gene (L110P/E148Q).

**Figure 1:**
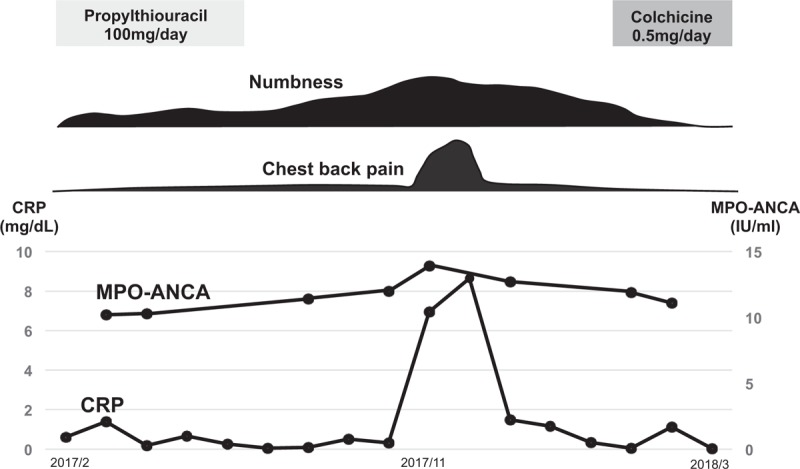
Clinical course. CRP = C-reactive protein, MPO-ANCA = myeloperoxidase antineutrophil cytoplasmic antibody.

After initiation of 0.5 mg/day colchicine, his numbness improved, and CRP levels declined to a normal range in March (Fig. 1). Fever did not recurrent after the administration of colchicine.

## 3 Discussion

We have described the successful treatment of a patient with ANCA-positive FMF whose numbness improved after the administration of colchicine.

ANCA production might be induced in patients with FMF. ANCA is a promising biomarker for AAV diagnosis, but is sometimes found in other diseases such as inflammatory bowel diseases, interstitial pneumonia, and infectious endocarditis.^[10–12]^ Although the mechanism of ANCA production is not fully understood, a previous report suggested that NETs are involved in ANCA production.^[4]^ NETs play an important role in defense against infection, and as chromatin in the nucleus released from activated neutrophils,^[13]^ exposure of intracellular antigens such as MPO might induce ANCA production.^[14]^ A recent report showed that neutrophils in peripheral blood of patients with FMF excrete NETs in the thermophilic phase,^[6]^ and other reports identify patients with ANCA-positive FMF.^[7,8]^ Therefore, ANCA production may also be induced in patients with FMF via NETs.

ANCA may have caused vasculitis in the present patient, and the administration of colchicine improved the neurological symptoms in this case. Although a previous report described neurological symptoms in patients with FMF, these were mainly caused by amyloidosis and were irreversible with poor therapeutic efficacy.^[15]^ On the other hand, nervous system manifestations are a common symptom in AAV.^[16]^ Interestingly, a previous report showed that PTU affected vasculitis development via disordered NETs.^[17]^ Therefore, patients with FMF who take PTU may develop small vessel vasculitis via disordered NETs.

In conclusion, the appearance of ANCA and vasculitis development should be considered in the clinical course of patients with FMF.

## Author contributions

**Conceptualization:** Sorato Segoe, Ken-ei Sada, Keigo Hayashi.

**Data curation:** Sorato Segoe, Ken-ei Sada, Keigo Hayashi.

**Project administration:** Ken-ei Sada.

**Supervision:** Ken-ei Sada, Jun Wada.

**Writing – original draft:** Sorato Segoe, Ken-ei Sada, Keigo Hayashi.

**Writing – review & editing:** Sorato Segoe, Ken-ei Sada, Yuriko Yamamura, Haruki Watanabe, Michiko Morishita, Yoshinori Matsumoto, Jun Wada.
